# Bis(2,1,3-benzoselenadiazole-κ*N*)dibromidocopper(II)

**DOI:** 10.1107/S160053681005422X

**Published:** 2011-01-15

**Authors:** Hoong-Kun Fun, Jia Hao Goh, Annada C. Maity, Shyamaprosad Goswami

**Affiliations:** a X-ray Crystallography Unit, School of Physics, Universiti Sains Malaysia, 11800 USM, Penang, Malaysia; b Department of Chemistry, Bengal Engineering and Science University, Shibpur, Howrah 711 103, India

## Abstract

In the title complex, [CuBr_2_(C_6_H_4_N_2_Se)_2_], the Cu^II^ ion is tetra­coordinated by two bromide anions and two N atoms in a distorted square-planar geometry. The two essentially planar 2,1,3-benzoselenadiazole ligands [maximum deviations = 0.012 (2) and 0.030 (2) Å] are approximately coplanar [dihedral angle = 6.14 (6)°]. In the crystal, short inter­molecular Se⋯Br, Se⋯N and N⋯N inter­actions are observed. These short inter­actions and inter­molecular C—H⋯Br hydrogen bonds link the complex mol­ecules into two-dimensional arrays parallel to the *ac* plane.

## Related literature

For general background to and applications of the title complex, see: Fun *et al.* (2008 ▶); Zhou *et al.* (2005 ▶). For related structures, see: Fun *et al.* (2008 ▶); Goswami *et al.* (2009 ▶). For the stability of the temperature controller used in the data collection, see: Cosier & Glazer (1986) ▶.
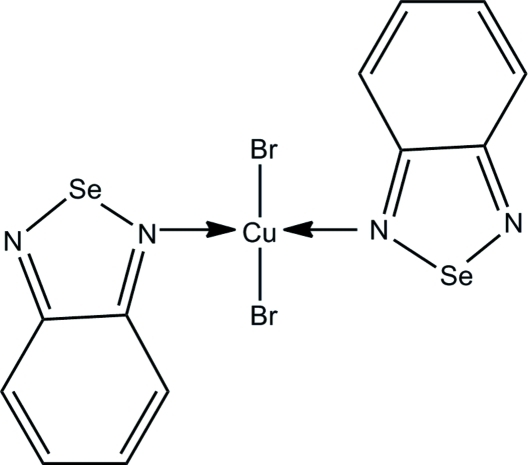


## Experimental

### 

#### Crystal data

[CuBr_2_(C_6_H_4_N_2_Se)_2_]
*M* *_r_* = 589.50
Triclinic, 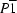

*a* = 8.3406 (1) Å
*b* = 9.5853 (1) Å
*c* = 10.2908 (1) Å
α = 94.627 (1)°
β = 109.640 (1)°
γ = 102.690 (1)°
*V* = 745.17 (1) Å^3^
*Z* = 2
Mo *K*α radiation
μ = 11.71 mm^−1^
*T* = 100 K
0.52 × 0.11 × 0.06 mm

#### Data collection

Bruker SMART APEXII CCD area-detector diffractometer
Absorption correction: multi-scan (*SADABS*; Bruker, 2009 ▶) *T*_min_ = 0.064, *T*_max_ = 0.550
47530 measured reflections
9041 independent reflections
6854 reflections with *I* > 2σ(*I*)
*R*_int_ = 0.048

#### Refinement

*R*[*F* ^2^ > 2σ(*F* ^2^)] = 0.030
*wR*(*F* ^2^) = 0.066
*S* = 1.02
9041 reflections
190 parameters
H-atom parameters constrained
Δρ_max_ = 0.96 e Å^−3^
Δρ_min_ = −1.28 e Å^−3^

### 

Data collection: *APEX2* (Bruker, 2009 ▶); cell refinement: *SAINT* (Bruker, 2009 ▶); data reduction: *SAINT*; program(s) used to solve structure: *SHELXTL* (Sheldrick, 2008 ▶); program(s) used to refine structure: *SHELXTL*; molecular graphics: *SHELXTL*; software used to prepare material for publication: *SHELXTL* and *PLATON* (Spek, 2009 ▶).

## Supplementary Material

Crystal structure: contains datablocks global, I. DOI: 10.1107/S160053681005422X/sj5081sup1.cif

Structure factors: contains datablocks I. DOI: 10.1107/S160053681005422X/sj5081Isup2.hkl

Additional supplementary materials:  crystallographic information; 3D view; checkCIF report

## Figures and Tables

**Table 1:** Selected interatomic distances (Å)

Cu1—N3	2.0077 (15)
Cu1—N1	2.0106 (15)
Cu1—Br1	2.3987 (3)
Cu1—Br2	2.4449 (3)
Se1⋯N2	1.7768 (15)
Se1⋯N1	1.8131 (16)
Se2⋯N4	1.7854 (15)
Se2⋯N3	1.8097 (15)
Se1⋯Br1^i^	3.5223 (3)
Se2⋯N2^ii^	2.6848 (16)
N2⋯N4^iii^	2.819 (2)

Symmetry codes: (i) 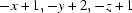; (ii) 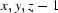; (iii) 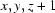.

**Table 2:** Hydrogen-bond geometry (Å, °)

*D*—H⋯*A*	*D*—H	H⋯*A*	*D*⋯*A*	*D*—H⋯*A*
C2—H2⋯Br2^iv^	0.93	2.74	3.464 (2)	135
C8—H8⋯Br1^i^	0.93	2.90	3.762 (2)	154

Symmetry codes: (i) 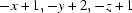; (iv) 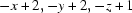.

**Fig. 1.**
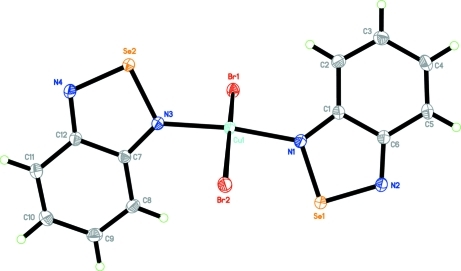
The molecular structure of the title complex, showing 50% probability displacement ellipsoids for non-H atoms and the atom-numbering scheme.

**Fig. 2.**
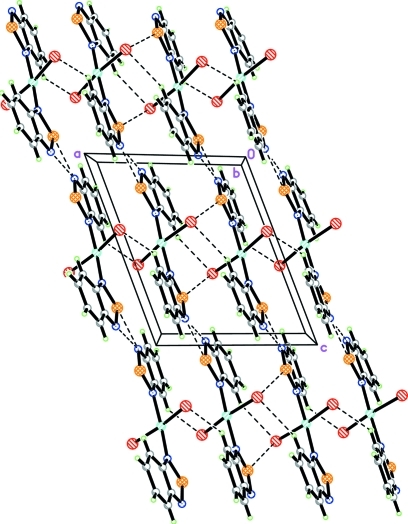
The crystal structure of the title complex, viewed along the b axis, showing a two-dimensional array parallel with the ac plane. Intermolecular interactions are shown as dashed lines.

## supplementary crystallographic information

### Comment

The design and synthesis of metal-organic framework (MOF) materials are an interesting area nowadays. The coordination chemistry of 2,1,3-benzoselenadiazole (bsd) has been sparingly explored (Zhou *et al.*, 2005). Recently we have shown that 2,1,3-bsd is capable of forming coordination networks with Zn(II) metal (Fun *et al.*, 2008). In the present work, we report the coordination networks for Cu^II^ complex containing bsd. Reaction of 2,1,3-bsd with CuBr_2_ results in the formation of the title copper complex.

The title complex comprises a neutral CuBr_2_*L*_2_ molecule (*L* = 2,1,3-bsd ligand). The Cu^II^ ion is tetra-coordinated by two Br^-^ ions and two *L* ligand-N atoms in a distorted square planar geometry, as indicated by angles of N1–Cu1–N3 = 172.82 (6)°, Br1–Cu1–Br2 = 170.661 (12)° and range of N–Cu–Br = 87.91 (5)–91.11 (5)°. The two ligands [(C1–C6/N1/N2/Se1) and (C6–C12/N3/N4/Se2)] are essentially planar, with maximum deviations of -0.012 (2) Å at atom C2 and 0.030 (2) Å at atom N3, respectively. These two ligands are approximately coplanar to one another, forming an interplanar angle of 6.14 (6)°. Selected bond lengths are listed in Table 1. All other bond lengths and angles are consistent to those observed in closely related structures (Fun *et al.*, 2008; Goswami *et al.*, 2009).

The interesting feature of the crystal packing is the observation of intermolecular short Se1···Br1, Se2···N2 and N2···N4 interactions (Table 1), as observed in the reported ZnCl_2_*L*_2_ structure (Fun *et al.*, 2008). The title complex is interconnected into two-dimensional arrays lying parallel with the *ac* plane *via* these short interactions as well as intermolecular C2—H2···Br2 and C8—H8···Br1 hydrogen bonds (Table 2).

### Experimental

A mixture of 2,1,3-bsd (1 g, 5.4 mol) and anhydrous copper bromide (606 mg, 2.72 mmol) in dry methanol (20 ml) was heated at 343–353 K for 2 h. After completion of the reaction, the mixture was allowed to cool to room temperature and the precipitate was collected by filtration. Recrystallization from methanol (25 %) in chloroform afforded brown microcrystalline solids of the title compound.

### Refinement

All aromatic-H atoms were placed in their calculated positions, with C—H = 0.93 Å, and refined using a riding model with *U*_iso_ = 1.2 *U*_eq_(C). The highest residual electron density peak is located at 0.71 Å from C11 and the deepest hole is located at 0.46 Å from Br2.

### Crystal data

**Table:**

[CuBr_2_(C_6_H_4_N_2_Se)_2_]	*Z* = 2
*M**_r_* = 589.50	*F*(000) = 550
Triclinic, *P*1	*D*_x_ = 2.627 Mg m^−^^3^
Hall symbol: -P 1	Mo *K*α radiation, λ = 0.71073 Å
*a* = 8.3406 (1) Å	Cell parameters from 9451 reflections
*b* = 9.5853 (1) Å	θ = 2.1–39.8°
*c* = 10.2908 (1) Å	µ = 11.71 mm^−^^1^
α = 94.627 (1)°	*T* = 100 K
β = 109.640 (1)°	Block, red
γ = 102.690 (1)°	0.52 × 0.11 × 0.06 mm
*V* = 745.17 (1) Å^3^	

### Data collection

**Table:**

Bruker SMART APEXII CCD area-detector diffractometer	9041 independent reflections
Radiation source: fine-focus sealed tube	6854 reflections with *I* > 2σ(*I*)
graphite	*R*_int_ = 0.048
φ and ω scans	θ_max_ = 39.8°, θ_min_ = 2.1°
Absorption correction: multi-scan (*SADABS*; Bruker, 2009)	*h* = −14→14
*T*_min_ = 0.064, *T*_max_ = 0.550	*k* = −17→17
47530 measured reflections	*l* = −18→18

### Refinement

**Table:**

Refinement on *F*^2^	Primary atom site location: structure-invariant direct methods
Least-squares matrix: full	Secondary atom site location: difference Fourier map
*R*[*F*^2^ > 2σ(*F*^2^)] = 0.030	Hydrogen site location: inferred from neighbouring sites
*wR*(*F*^2^) = 0.066	H-atom parameters constrained
*S* = 1.02	*w* = 1/[σ^2^(*F*_o_^2^) + (0.025*P*)^2^ + 0.1897*P*] where *P* = (*F*_o_^2^ + 2*F*_c_^2^)/3
9041 reflections	(Δ/σ)_max_ = 0.003
190 parameters	Δρ_max_ = 0.96 e Å^−^^3^
0 restraints	Δρ_min_ = −1.28 e Å^−^^3^

### Special details

**Table:**

Experimental. The crystal was placed in the cold stream of an Oxford Cryosystems Cobra open-flow nitrogen cryostat (Cosier & Glazer, 1986) operating at 100.0 (1)K.
Geometry. All esds (except the esd in the dihedral angle between two l.s. planes) are estimated using the full covariance matrix. The cell esds are taken into account individually in the estimation of esds in distances, angles and torsion angles; correlations between esds in cell parameters are only used when they are defined by crystal symmetry. An approximate (isotropic) treatment of cell esds is used for estimating esds involving l.s. planes.
Refinement. Refinement of F^2^ against ALL reflections. The weighted R-factor wR and goodness of fit S are based on F^2^, conventional R-factors R are based on F, with F set to zero for negative F^2^. The threshold expression of F^2^ > 2sigma(F^2^) is used only for calculating R-factors(gt) etc. and is not relevant to the choice of reflections for refinement. R-factors based on F^2^ are statistically about twice as large as those based on F, and R- factors based on ALL data will be even larger.

### Fractional atomic coordinates and isotropic or equivalent isotropic displacement parameters (Å^2^)

**Table:**

	*x*	*y*	*z*	*U*_iso_*/*U*_eq_	
Cu1	0.76323 (3)	0.96053 (2)	0.46923 (2)	0.01342 (4)	
Se1	0.76418 (3)	1.00702 (2)	0.790290 (19)	0.01462 (4)	
Se2	0.76370 (3)	0.96029 (2)	0.148426 (18)	0.01419 (4)	
Br1	0.50628 (3)	0.76430 (2)	0.346275 (19)	0.01659 (4)	
Br2	0.99180 (3)	1.18054 (2)	0.602981 (19)	0.01630 (4)	
N1	0.7843 (2)	0.89074 (17)	0.65184 (16)	0.0145 (3)	
N2	0.7949 (2)	0.87760 (18)	0.90445 (16)	0.0157 (3)	
N3	0.7309 (2)	1.05001 (17)	0.29546 (16)	0.0145 (3)	
N4	0.7204 (2)	1.10188 (18)	0.05009 (16)	0.0156 (3)	
C1	0.8108 (2)	0.7679 (2)	0.69755 (18)	0.0141 (3)	
C2	0.8325 (3)	0.6474 (2)	0.6210 (2)	0.0168 (3)	
H2	0.8267	0.6476	0.5292	0.020*	
C3	0.8621 (3)	0.5318 (2)	0.6854 (2)	0.0188 (3)	
H3	0.8784	0.4535	0.6365	0.023*	
C4	0.8690 (3)	0.5269 (2)	0.8263 (2)	0.0188 (3)	
H4	0.8888	0.4456	0.8660	0.023*	
C5	0.8472 (3)	0.6385 (2)	0.9022 (2)	0.0180 (3)	
H5	0.8509	0.6343	0.9931	0.022*	
C6	0.8185 (2)	0.7628 (2)	0.83934 (19)	0.0149 (3)	
C7	0.6893 (2)	1.1727 (2)	0.26414 (18)	0.0139 (3)	
C8	0.6457 (3)	1.2712 (2)	0.34984 (19)	0.0168 (3)	
H8	0.6437	1.2531	0.4368	0.020*	
C9	0.6071 (3)	1.3927 (2)	0.3011 (2)	0.0188 (3)	
H9	0.5777	1.4572	0.3560	0.023*	
C10	0.6102 (3)	1.4242 (2)	0.1680 (2)	0.0199 (4)	
H10	0.5866	1.5097	0.1401	0.024*	
C11	0.6472 (3)	1.3311 (2)	0.0817 (2)	0.0181 (3)	
H11	0.6471	1.3515	−0.0052	0.022*	
C12	0.6860 (2)	1.2015 (2)	0.12748 (18)	0.0147 (3)	

### Atomic displacement parameters (Å^2^)

**Table:**

	*U*^11^	*U*^22^	*U*^33^	*U*^12^	*U*^13^	*U*^23^
Cu1	0.01609 (10)	0.01305 (10)	0.01093 (9)	0.00325 (8)	0.00478 (7)	0.00314 (7)
Se1	0.01835 (8)	0.01413 (8)	0.01224 (7)	0.00580 (6)	0.00550 (6)	0.00300 (6)
Se2	0.01791 (8)	0.01411 (8)	0.01127 (7)	0.00550 (6)	0.00527 (6)	0.00277 (6)
Br1	0.01675 (8)	0.01600 (8)	0.01488 (7)	0.00238 (6)	0.00416 (6)	0.00318 (6)
Br2	0.01769 (8)	0.01514 (8)	0.01563 (8)	0.00415 (6)	0.00532 (6)	0.00391 (6)
N1	0.0175 (7)	0.0141 (7)	0.0120 (6)	0.0036 (5)	0.0058 (5)	0.0027 (5)
N2	0.0185 (7)	0.0179 (7)	0.0124 (6)	0.0071 (6)	0.0059 (5)	0.0043 (5)
N3	0.0179 (7)	0.0137 (7)	0.0120 (6)	0.0041 (5)	0.0057 (5)	0.0023 (5)
N4	0.0186 (7)	0.0175 (7)	0.0128 (6)	0.0071 (6)	0.0065 (5)	0.0050 (5)
C1	0.0147 (7)	0.0142 (7)	0.0134 (7)	0.0039 (6)	0.0048 (6)	0.0039 (6)
C2	0.0196 (8)	0.0158 (8)	0.0155 (7)	0.0042 (7)	0.0076 (7)	0.0016 (6)
C3	0.0209 (9)	0.0156 (8)	0.0191 (8)	0.0053 (7)	0.0062 (7)	0.0010 (6)
C4	0.0200 (9)	0.0163 (8)	0.0194 (8)	0.0059 (7)	0.0048 (7)	0.0067 (7)
C5	0.0220 (9)	0.0176 (8)	0.0157 (7)	0.0069 (7)	0.0065 (7)	0.0072 (6)
C6	0.0154 (7)	0.0158 (8)	0.0140 (7)	0.0044 (6)	0.0053 (6)	0.0036 (6)
C7	0.0145 (7)	0.0136 (7)	0.0130 (7)	0.0037 (6)	0.0041 (6)	0.0024 (6)
C8	0.0189 (8)	0.0191 (9)	0.0129 (7)	0.0064 (7)	0.0056 (6)	0.0017 (6)
C9	0.0198 (9)	0.0196 (9)	0.0175 (8)	0.0080 (7)	0.0061 (7)	0.0014 (7)
C10	0.0230 (9)	0.0178 (9)	0.0204 (8)	0.0086 (7)	0.0072 (7)	0.0060 (7)
C11	0.0221 (9)	0.0179 (8)	0.0160 (7)	0.0076 (7)	0.0069 (7)	0.0061 (6)
C12	0.0159 (7)	0.0153 (8)	0.0131 (7)	0.0042 (6)	0.0052 (6)	0.0034 (6)

### Geometric parameters (Å, °)

**Table:**

Cu1—N3	2.0077 (15)	C3—C4	1.437 (3)
Cu1—N1	2.0106 (15)	C3—H3	0.9300
Cu1—Br1	2.3987 (3)	C4—C5	1.354 (3)
Cu1—Br2	2.4449 (3)	C4—H4	0.9300
Se1—N2	1.7768 (15)	C5—C6	1.429 (3)
Se1—N1	1.8131 (16)	C5—H5	0.9300
Se2—N4	1.7854 (15)	C7—C8	1.426 (3)
Se2—N3	1.8097 (15)	C7—C12	1.448 (2)
N1—C1	1.339 (2)	C8—C9	1.365 (3)
N2—C6	1.328 (2)	C8—H8	0.9300
N3—C7	1.331 (2)	C9—C10	1.433 (3)
N4—C12	1.334 (2)	C9—H9	0.9300
C1—C2	1.424 (3)	C10—C11	1.363 (3)
C1—C6	1.444 (2)	C10—H10	0.9300
C2—C3	1.362 (3)	C11—C12	1.428 (3)
C2—H2	0.9300	C11—H11	0.9300
			
Cu1···N3	2.0077 (15)	Se2···N4	1.7854 (15)
Cu1···N1	2.0106 (15)	Se2···N3	1.8097 (15)
Cu1···Br1	2.3987 (3)	Se1···Br1^i^	3.5223 (3)
Cu1···Br2	2.4449 (3)	Se2···N2^ii^	2.6848 (16)
Se1···N2	1.7768 (15)	N2···N4^iii^	2.819 (2)
Se1···N1	1.8131 (16)		
			
N3—Cu1—N1	172.82 (6)	C5—C4—H4	119.4
N3—Cu1—Br1	90.80 (5)	C3—C4—H4	119.4
N1—Cu1—Br1	91.11 (5)	C4—C5—C6	118.41 (17)
N3—Cu1—Br2	89.08 (5)	C4—C5—H5	120.8
N1—Cu1—Br2	87.91 (5)	C6—C5—H5	120.8
Br1—Cu1—Br2	170.661 (12)	N2—C6—C5	123.19 (17)
N2—Se1—N1	92.13 (7)	N2—C6—C1	116.30 (16)
N4—Se2—N3	92.13 (7)	C5—C6—C1	120.50 (17)
C1—N1—Se1	108.34 (12)	N3—C7—C8	126.26 (16)
C1—N1—Cu1	132.71 (13)	N3—C7—C12	114.19 (16)
Se1—N1—Cu1	118.95 (8)	C8—C7—C12	119.53 (16)
C6—N2—Se1	108.82 (12)	C9—C8—C7	118.33 (17)
C7—N3—Se2	108.91 (12)	C9—C8—H8	120.8
C7—N3—Cu1	131.38 (13)	C7—C8—H8	120.8
Se2—N3—Cu1	119.70 (8)	C8—C9—C10	122.20 (18)
C12—N4—Se2	108.23 (12)	C8—C9—H9	118.9
N1—C1—C2	126.44 (16)	C10—C9—H9	118.9
N1—C1—C6	114.38 (16)	C11—C10—C9	121.24 (18)
C2—C1—C6	119.18 (16)	C11—C10—H10	119.4
C3—C2—C1	118.50 (17)	C9—C10—H10	119.4
C3—C2—H2	120.7	C10—C11—C12	118.52 (17)
C1—C2—H2	120.7	C10—C11—H11	120.7
C2—C3—C4	122.18 (18)	C12—C11—H11	120.7
C2—C3—H3	118.9	N4—C12—C11	123.37 (17)
C4—C3—H3	118.9	N4—C12—C7	116.52 (16)
C5—C4—C3	121.22 (18)	C11—C12—C7	120.11 (17)
			
N2—Se1—N1—C1	0.24 (14)	Se1—N2—C6—C1	−1.1 (2)
N2—Se1—N1—Cu1	179.82 (10)	C4—C5—C6—N2	179.72 (19)
Br1—Cu1—N1—C1	61.95 (18)	C4—C5—C6—C1	0.7 (3)
Br2—Cu1—N1—C1	−127.35 (18)	N1—C1—C6—N2	1.3 (3)
Br1—Cu1—N1—Se1	−117.52 (8)	C2—C1—C6—N2	−179.12 (17)
Br2—Cu1—N1—Se1	53.19 (8)	N1—C1—C6—C5	−179.62 (18)
N1—Se1—N2—C6	0.48 (14)	C2—C1—C6—C5	−0.1 (3)
N4—Se2—N3—C7	0.87 (14)	Se2—N3—C7—C8	177.08 (16)
N4—Se2—N3—Cu1	−178.25 (10)	Cu1—N3—C7—C8	−3.9 (3)
Br1—Cu1—N3—C7	112.79 (17)	Se2—N3—C7—C12	−1.4 (2)
Br2—Cu1—N3—C7	−57.87 (17)	Cu1—N3—C7—C12	177.57 (13)
Br1—Cu1—N3—Se2	−68.31 (9)	N3—C7—C8—C9	179.67 (19)
Br2—Cu1—N3—Se2	121.03 (9)	C12—C7—C8—C9	−1.9 (3)
N3—Se2—N4—C12	−0.07 (14)	C7—C8—C9—C10	−0.4 (3)
Se1—N1—C1—C2	179.64 (16)	C8—C9—C10—C11	2.0 (3)
Cu1—N1—C1—C2	0.1 (3)	C9—C10—C11—C12	−1.0 (3)
Se1—N1—C1—C6	−0.9 (2)	Se2—N4—C12—C11	179.34 (16)
Cu1—N1—C1—C6	179.63 (13)	Se2—N4—C12—C7	−0.7 (2)
N1—C1—C2—C3	178.66 (19)	C10—C11—C12—N4	178.62 (19)
C6—C1—C2—C3	−0.8 (3)	C10—C11—C12—C7	−1.3 (3)
C1—C2—C3—C4	1.1 (3)	N3—C7—C12—N4	1.5 (3)
C2—C3—C4—C5	−0.4 (3)	C8—C7—C12—N4	−177.12 (17)
C3—C4—C5—C6	−0.5 (3)	N3—C7—C12—C11	−178.57 (18)
Se1—N2—C6—C5	179.91 (16)	C8—C7—C12—C11	2.8 (3)

Symmetry codes: (i) −*x*+1, −*y*+2, −*z*+1; (ii) *x*, *y*, *z*−1; (iii) *x*, *y*, *z*+1.

### Hydrogen-bond geometry (Å, °)

**Table:**

*D*—H···*A*	*D*—H	H···*A*	*D*···*A*	*D*—H···*A*
C2—H2···Br2^iv^	0.93	2.74	3.464 (2)	135
C8—H8···Br1^i^	0.93	2.90	3.762 (2)	154

Symmetry codes: (iv) −*x*+2, −*y*+2, −*z*+1; (i) −*x*+1, −*y*+2, −*z*+1.
